# Improving Staff Awareness of Sensory Aid Needs and Dementia Status on an Old Age Ward

**DOI:** 10.1192/bjo.2024.343

**Published:** 2024-08-01

**Authors:** Bekim Arifaj, Limaro Nyam, Karen Sansom-Ninnes

**Affiliations:** King's College London, London, United Kingdom

## Abstract

**Aims:**

The aim of our quality improvement project was to explore and improve care for patients who use sensory aids, with or without dementia, on an old age ward at King's College Hospital. We sought to do this by increasing the staff awareness of each patient's sensory needs and dementia status.

Guidelines state that sensory aids (glasses and hearing aids) are important in orientating patients with delirium and dementia, yet these devices frequently go missing during admission or are not being used appropriately. This could affect communication and therefore overall care, both physical and mental. It is widely understood that delirium and dementia are associated with increased morbidity and mortality. In this project we aimed to explore issues around sensory aid use and to identify and implement impactful changes.

**Methods:**

2 Plan, Do, Study, Act cycles were conducted between October 2022 to February 2023. A driver diagram was created following staff interviews on the ward. The first cycle focused on increasing awareness of a form in electronic patient records (EPR) and the need for documenting each patient's sensory aid possessions and dementia status. This was done through bite-size teaching sessions to the team and monitoring of completion of this form. The second cycle included utilising a new laminated bedside checklist that is manually filled in and was aimed to serve as a visual cue of the patient's sensory impairment/dementia status. A survey was used at baseline and then repeated over the course of both cycles to evaluate awareness of staff (named nurse) of each patient's sensory impairment/dementia status on the ward.

**Results:**

Baseline survey showed that staff were unsure of the sensory aid needs (glasses, hearing aids, dentures) of 25% of patients and 46.7% when it came to dementia status. EPR form completion increased by 14% between 14/12/22 and 25/01/23, however this was not statistically significant. 18% of bedside checklists were filled after 4 weeks. Overall, there was a statistically significant decrease in staff not knowing the sensory impairment status (by 32%) as well as dementia status (by 40%).

**Conclusion:**

Whilst uptake of the forms and bedside checklist was slow, the project did show an improvement in awareness of staff and our hypothesis is that this leads to better use of sensory aids. The next step would be to assess whether this leads to better care through further PDSA cycles.

